# Open-Label Single-Sequence Crossover Study Evaluating Pharmacokinetics, Efficacy, and Safety of Once-Daily Dosing of Nitisinone in Patients with Hereditary Tyrosinemia Type 1

**DOI:** 10.1007/8904_2017_29

**Published:** 2017-06-23

**Authors:** Nathalie Guffon, Anders Bröijersén, Ingrid Palmgren, Mattias Rudebeck, Birgitta Olsson

**Affiliations:** 13 Centre de Référence des Maladies Héréditaires du Métabolisme, Hôpital Femme Mère Enfant, 59 Boulevard Pinel, 69677 Bron, France; 14 Swedish Orphan Biovitrum (Sobi), 112 76 Stockholm, Sweden; 1 Div Metabolism & Childrens Res Ctr, University Childrens Hospital, Zurich, Switzerland; 2 Div of Child and Adolescent Neurology, Mayo Clinic, Rochester, Minnesota USA; 3 Genetics & Genomic Med Prog, GOSHCC, UCL Institute of Child Health, London, United Kingdom; 4 Managing Editor JIMD, Univ Children´s Hospital Heidelberg, Heidelberg, Germany; 5 Hayward Genetics Ctr,SL#31, Tulane Univ Medical Health Ctr, New Orleans, Louisiana USA; 6 Division of Human Genetics, Medical University Innsbruck, Innsbruck, Austria

## Abstract

*Background*: Although nitisinone is successfully used to treat hereditary tyrosinemia type 1 (HT-1) with the recommended twice-daily dosing, data describing a long half-life motivate less frequent dosing. Therefore, in agreement with the Pharmacovigilance Risk Assessment Committee at the European Medicines Agency, this study was performed to investigate the switch to once-daily dosing.

*Methods*: This open-label, non-randomized, single-sequence crossover study evaluated the pharmacokinetics, efficacy, and safety of once-daily compared to twice-daily dosing of nitisinone in patients with HT-1 (NCT02323529). Well-controlled patients of <2, 2 to <12, 12 to <18, and ≥18 years of age who were on twice-daily dosing were eligible for participation. Nitisinone and succinylacetone levels were determined from dry blood spots by tandem mass spectrometry. The primary endpoint was C_min_ of nitisinone after ≥4 weeks of treatment on each dosing regimen. Secondary objectives were evaluation of efficacy and safety during each dosing regimen.

*Results*: In total, 19 patients were enrolled and 17 included in the per-protocol analysis set. The mean (SD) nitisinone C_min_ decreased by 23%, from 26.4 (10.2) to 21.2 (9.9) μmol/L in dry blood spot samples (not equivalent to plasma concentrations), when patients switched from twice- to once-daily dosing. There was no apparent age- or bodyweight-related trend in the degree of C_min_ decrease. No patient had quantifiable succinylacetone levels during the once-daily treatment period, indicating efficacious treatment. All adverse events were mild or moderate and judged unrelated to nitisinone.

*Conclusion*: The switch to once-daily treatment with nitisinone appeared efficacious and safe in the treatment of patients with HT-1.

## Introduction

Hereditary tyrosinemia type 1 (HT-1, OMIM reference 276700) is a rare metabolic disorder with an incidence of 1 in 100,000 worldwide (Hutchesson et al. 1996). The disease is caused by mutations in the *FAH* gene causing defects in fumarylacetoacetate hydrolase (FAH, EC 3.7.1.2), the final enzyme of the pathway responsible for degradation of tyrosine. As a consequence, the catabolic toxic intermediates maleylacetoacetate and fumarylacetoacetate accumulate and convert into succinylacetone (SA) and succinylacetoacetate causing liver damage including hepatocellular carcinoma as well as kidney dysfunction, neurological problems, and shorter life expectancy (Lindblad et al. 1977; Mitchell et al. 2001; van Ginkel et al. 2016).

Nitisinone, also known as NTBC (Orfadin^®^, Sobi), is a reversible inhibitor of 4-hydroxyphenylpyruvate dioxygenase (HPPD, EC 1.13.11.27), an enzyme upstream of FAH in the tyrosine catabolic pathway that prevents accumulation of toxic metabolites (Schulz et al. 1993). To date, nitisinone is the only approved substance for the treatment of HT-1, and in combination with a low-tyrosine diet and special amino acid supplements, the treatment has resulted in a greater than 90% survival rate of patients with HT-1 (Larochelle et al. 2012). The drug is well tolerated with few side effects and the only alternative treatment option is liver transplantation. Early diagnosis is important to allow early treatment initiation and better long-term outcome and is facilitated in many countries by newborn screening, ideally using SA as a disease marker (De Jesus et al. 2014; Mayorandan et al. 2014).

The clinical study upon which the marketing approval of nitisinone was based practiced twice-daily dosing (Holme and Lindstedt 2000), which also appears as the most commonly practiced dosing frequency (Mayorandan et al. 2014). The long half-life in plasma, median 54 h (range: 39–86 h), has however motivated some clinicians to reduce dosing frequency to once-daily (Hall et al. 2001; McKiernan 2013). Moreover, once-daily dosing is advised in recent recommendations (de Laet et al. 2013), but the suitability of switching from twice- to once-daily dosing has not been properly documented; there is, however, one small study including nine patients reporting that once-daily dosing may be as effective as a multiple-dose regimen (Schlune et al. 2012). It was therefore agreed with the Pharmacovigilance Risk Assessment Committee (PRAC) at the European Medicines Agency (EMA) to perform the study presented here, with the purpose of investigating the effect on nitisinone serum concentrations and clinical outcome, when switching patients of all ages with HT-1 to the less frequent once-daily dosing regimen, which may be preferable from a convenience and compliance perspective (Iskedjian et al. 2002; Coleman et al. 2012).

## Subjects and Methodology

### Subjects

Patients eligible for the study were male or female patients of all ages diagnosed with HT-1 who were well controlled on twice-daily, or more frequent, nitisinone dosing according to the investigator, and who had stable laboratory values: alkaline phosphatase, alanine aminotransferase, aspartate aminotransferase, bilirubin, and international normalized ratio. Women of childbearing age were to use contraception to allow study inclusion. Individuals with prior periods of once-daily dosing were excluded due to the risk of selection bias. Additional reasons for exclusion were: participation in any other interventional clinical study within 3 months prior to inclusion in this study, pregnancy, breast feeding, previous liver transplant, or patients who within the past 4 weeks prior to inclusion started any new medication for a previously undiagnosed illness, any foreseeable inability to cooperate with given instructions or study procedures, or any medical condition that in the opinion of the investigator made the patient unsuitable for inclusion. The first patient was included in December 2014 and the last patient’s last visit was in September 2015.

The study (www.clinicaltrials.gov identifier: NCT02323529) was conducted according to International Conference on Harmonisation-Good Clinical Practice guidelines and the Declaration of Helsinki and was approved by relevant regulatory authorities and independent ethics committees. Informed consent was obtained from the patient or patient’s legal representative (for patients under the age of 18) prior to any study intervention.

### Study Design

**Fig. 1:**
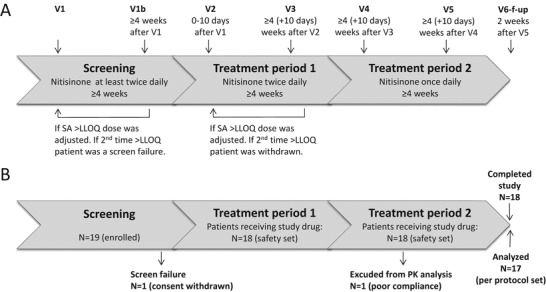
Study design and patient disposition. (**a**) Study design. (**b**) Patient disposition during the study

This was an open-label, non-randomized, single-sequence crossover study aiming to enroll a minimum of 20 patients with preferably 5 patients, but minimum 3 patients, in each age group (infants: <2 years of age, children: 2 years to <12 years of age, adolescents: 12 years to <18 years of age, adults: ≥18 years of age). Patients were enrolled from six sites in Belgium, Denmark, France, Germany, and Sweden. The study was divided into three periods: screening, treatment period 1, and treatment period 2 (Fig. 1a). At screening, if SA was quantifiable in urine or serum samples taken locally or in case of other signs of inadequate dosing, the patient’s nitisinone dose was to be adjusted and screening was repeated (maximum one time). Otherwise, the patient started treatment period 1 during which nitisinone was dosed twice-daily for at least 4 weeks. If SA was quantifiable at the end of treatment period 1, an adjusted higher dose was used for an additional 4 weeks, and if the SA levels were still quantifiable by the end of this period the patient was to be withdrawn from the study. Patients with no quantifiable SA, or no other signs of inadequate dosing, at the end of treatment period 1 continued to treatment period 2, during which nitisinone was dosed once-daily for at least 4 weeks.

### Study Intervention and Pharmacokinetic Assessment

Capsules of 2, 5, or 10 mg nitisinone were provided for the study. The individual nitisinone dose was the one prescribed by the treating physician during the screening period. The mode of administration (swallowing the capsules whole or mixing the contents with food or drinks) matched each patients’ prior habits and was noted in the case report form. Once patients entered treatment period 2 (once-daily dosing), the dose was taken in the morning. Considering the involvement of pediatric patients, blood sampling (volumes and occasions) was kept to a minimum and only C_min_ (minimum concentration) and C_max_ (maximum concentration) were studied, as these were the only two variables affected by a change in dosing frequency. Thus no full PK evaluation was performed. C_min_ was determined in samples taken immediately before dosing and because determination of C_max_ would have required blood sampling over several hours, a near maximum concentration was determined from a sample taken in the interval between 3 and 4 h after dosing. The choice of this sampling time was based on data from the only study with PK data for nitisinone at steady-state. That study, however, used a liquid formulation of nitisinone (Olsson et al. 2015). Samples for both C_min_ and C_max_ were taken at the end of each treatment period, i.e., at visits 3 and 5 (Fig. 1a).

### Outcome Measures

The primary study objective was to evaluate the steady-state exposure to nitisinone during once- and twice-daily dosing by assessing the C_min_ after at least 4 weeks of treatment on each dosing regimen, defined as the concentration in the dry blood spot (DBS) sample taken immediately before dosing. Secondary endpoints related to the primary objective were assessment of C_max_ of nitisinone and the C_max_/C_min_ ratio after at least 4 weeks of treatment on each dosage regimen. Additional secondary objectives were to evaluate the efficacy of nitisinone during once-daily dosing by assessing concentrations of SA after at least 4 weeks of treatment and nitisinone levels and C_min_ if SA was above the lower limit of quantification (LLOQ). An analytical method for the determination of nitisinone and SA in DBS, using liquid extraction followed by mass spectrometry (LC-MS/MS), was validated over concentration ranges of 0.500–120 μmol/L (nitisinone) and 0.250–50.0 μmol/L (SA). The limit of quantification in our assay was 0.250 μmol/L SA which was considered sufficiently low with regard to normal DBS SA levels measuring up to 1 μmol/L or more (Allard et al. 2004; la Marca et al. 2008; Turgeon et al. 2008; Dhillon et al. 2011; Yang et al. 2017). Central laboratory measurement of nitisinone and SA levels were made in the DBSs from visits 1 (only SA), 3, and 5. A conversion of whole blood (DBS) concentrations to serum concentrations, taking individual hematocrit values into account, was originally planned for both nitisinone and SA. This was, however, not performed because factors other than hematocrit values could influence these results and the method was not validated to allow such conversion. Moreover, for SA a conversion was not applicable since all concentrations in the DBS samples were below the LLOQ. For nitisinone, the actual serum concentration can be estimated to be approximately 1.6 times higher than the DBS concentrations (Sander et al. 2011).

In addition to the DBS samples that were sent to a central laboratory at the end of the study, blood (serum/plasma/DBS) or urine samples were tested in local laboratories according to local routine with the purpose of evaluating whether dose adjustments were necessary during the course of the study.

### Safety Assessments

Evaluation of safety during once- and twice-daily dosing was also included as a secondary objective. This was assessed by collection of adverse events (AEs), routine clinical chemistry tests including serum alpha fetoprotein, hepatic and renal function, coagulation, and serum tyrosine. All enrolled patients who received at least one dose of study drug were included in the safety analysis set. AEs were collected until the last visit whether or not the event was considered to be treatment related and serious adverse events (SAEs) were collected until 28 days past the last dose.

### Statistics

Due to the low prevalence of HT-1, the sample size was based on feasibility rather than on statistical power considerations. A minimum enrollment of 20 patients was planned.

All data were summarized descriptively. In addition, for the PK data, the geometric mean, associated 95% CI (confidence interval) for C_min_ and C_max_ and the C_max_/C_min_ ratio were calculated. Statistical analyses were performed using SAS software Version 9.1 or later (SAS Institute, Inc. Cary, North Carolina, USA).

## Results

### Demographics and Baseline Characteristics

In total, 19 patients were enrolled in the study. However, one patient withdrew her consent before entering treatment period 1, and one patient was excluded from the PK analysis due to poor compliance in the once-daily period, leaving 18 patients in the safety-set and 17 patients in the per-protocol set (Fig. 1b). The mean (SD) age was 13.2 (7.1) years, and ranged from 1.3 to 24.0 years. Due to recruitment difficulties, only two patients were included in the youngest age group while five to six patients were included in the other age groups. The gender distribution was equal (Table 1).

**Table 1:** Demographics and baseline characteristics (safety set)

	<2 years (*N* = 2)	2 to <12 years (*N* = 5)	12 to <18 years (*N* = 5)	≥18 years (*N* = 6)	All (*N* = 18)
*Age (years)*					
Mean (SD)	1.5 (0.2)	7.7 (2.4)	13.9 (1.2)	21.1 (2.3)	13.2 (7.1)
Min, max	1.3, 1.6	5.0, 11.0	13.0, 15.4	19.0, 24.0	1.3, 24.0
*Sex*					
Male	2 (100.0%)	4 (80.0%)	2 (40.0%)	1 (16.7%)	9 (50.0%)
Female	0	1 (20.0%)	3 (60.0%)	5 (83.3%)	9 (50.0%)
*Age at HT-1 diagnosis (months)*					
Mean (SD)	8.0 (2.1)	8.2 (6.4)	7.0 (4.1)	8.8 (5.3)	8.1 (4.8)
*Age at nitisinone treatment start (months)*					
Mean (SD)	8.5 (2.1)	8.2 (6.4)	7.2 (4.3)	15.3 (14.3)	10.3 (9.4)

*SD* standard deviation

### Nitisinone Exposure

**Fig. 2:**
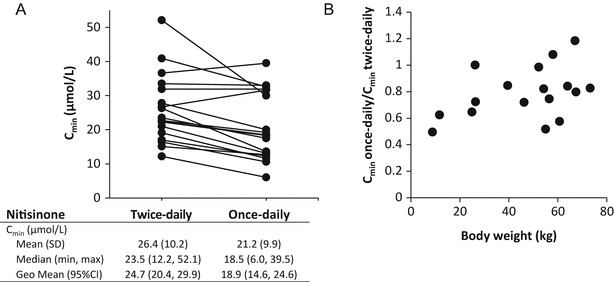
Nitisinone exposure (C_min_) after twice- and once-daily dosing in patients with HT-1. (**a**) Spaghetti plot illustrating C_min_ during twice- and once-daily dosing, *N* = 17. (**b**) Individual geometric mean treatment ratios of C_min_ once-daily/C_min_ twice-daily. Each *dot* represents one patient, *N* = 17. *CI* confidence interval, *C*_*min*_ minimum concentration, defined as pre-dose concentration, *CV* coefficient of variation, *SD* standard deviation

The nitisinone steady-state exposure during once- and twice-daily dosing was estimated by assessing the C_min_ and C_max_ values. The mean (SD) nitisinone C_min_ was lower (21.2 [9.9] μmol/L) for the once-daily treatment period compared to the twice-daily treatment period (26.4 [10.2] μmol/L) (Fig. 2a). All age groups showed a similar trend of lower C_min_ during once-daily compared to twice-daily dosing (not shown). The geometric mean treatment ratio (C_min_ once daily/C_min_ twice daily) was 0.77 (95% CI: 0.68, 0.87), corresponding to a 23% decrease in C_min_ after switching from twice- to once-daily dosing. Note that these concentrations assessed from DBS samples should not be directly compared to the serum concentrations mentioned in the treatment recommendations (de Laet et al. 2013).

The mean (SD) C_max_ was similar for the twice- (31.1 [14.3] μmol/L) and once-daily (29.3 [11.6] μmol/L) treatment period. However, it should be noted that the exact C_max_ was not determined in this study. For drugs like nitisinone with a linear relationship between dose and plasma concentration (linear pharmacokinetics) a change in dosing frequency with maintained total dose does not change the overall average drug concentration in the dosage interval but rather the fluctuations around the average. Once-daily dosing was therefore expected to result in an increase in C_max_ of the same magnitude as the decrease in C_min_ compared to twice-daily dosing. For this reason these results indicate that for many patients the optimum sampling time for determination of C_max_ was not within 3–4 h after dosing, since in general no such increase was observed.

The once-daily/twice-daily C_min_ ratios varied among patients and plotting the data by age, instead of bodyweight, provided overall similar results (Fig. 2b and not shown). Due to the low number of patients and the fact that some patients with higher weight (also older) had similar ratios as the patient with the lowest weight (youngest), it can be concluded that the once-daily/twice-daily C_min_ ratios appeared to be independent of patient weight and age.

No patient required a dose adjustment after switching to once daily dosing. Thus, the mean (SD) prescribed daily nitisinone dose was the same, 0.78 (0.27) mg/kg (*N* = 18), for both the twice-daily and the once-daily dosing periods (not shown).

### Efficacy of Nitisinone Treatment

Since quantifiable levels of SA indicate insufficient inhibition of HPPD, treatment efficacy was determined by the proportion of patients with quantifiable serum or urine SA levels as assessed by both local and central laboratory, after at least 4 weeks of once-daily nitisinone treatment. No patient had SA levels above the LLOQ at the end of the once-daily treatment period. However, in a local plasma sample at the end of the twice-daily treatment period, one patient had SA levels above LLOQ. After 4 weeks on an increased dose, and no detectable SA, the patient entered the once-daily period.

### Safety

Overall, 15 patients (83.3%) experienced at least 1 AE during the study (Table 2). Thirteen patients (72.2%) experienced at least 1 AE during the twice-daily treatment period and 11 patients (61.1%) experienced at least 1 AE during the once-daily treatment period. All AEs were mild or moderate in intensity. One patient experienced an SAE (gastroenteritis) during the twice-daily treatment period. No AE (including the SAE) was considered by the investigator to be related to the nitisinone treatment. No patient had any clinically significant change in any laboratory parameter, including serum tyrosine levels, or an AE associated with a safety laboratory parameter.

**Table 2:** Number of adverse events (AEs) (safety set)

Category	Twice-daily treatment period (*N* = 18)	Once-daily treatment period (*N* = 18)	Any treatment period (*N* = 18)
*Patients who had an AE*	13 (72.2%)	11 (61.1%)	15 (83.3%)
Number of AEs	18	18	36
*Patient who had a mild AE*	12 (66.7%)	11 (61.1%)	14 (77.8%)
Number of mild AEs	15	16	31
*Patients who had a moderate AE*	2 (11.1%)	2 (11.1%)	4 (22.2%)
Number of moderate AEs	3	2	5
*Patients who had a severe AE*	0	0	0
*Patients who had a treatment-related AE* ^a^	0	0	0
*Patients who had an SAE*	1 (5.6%)	0	1 (5.6%)
Number of SAEs	1	0	1
*Patients who had an AE resulting in death*	0	0	0

Note: Only AEs after first dose of study drug included. Percentages based on the number of patients in each treatment period

*AE* adverse event, *SAE* serious adverse event

^a^Relationship judged by the investigator

Most AEs were within the system organ class “infections and infestations”; 8 patients with 8 events in the twice-daily treatment period and 5 patients with 5 events in the once-daily treatment period. In conclusion there were no apparent differences in either the number or the type of AEs between twice- and once-daily dosing periods.

## Discussion

In this prospective single-sequence crossover study on 18 patients with HT-1 that compared the efficacy, safety, and steady-state exposure of nitisinone during once- and twice-daily dosing, once-daily treatment appeared as efficacious and safe as twice-daily treatment. There were no clinically significant differences in the number or type of AEs between the treatment regimens.

To our knowledge there are no clinical studies to date investigating the optimal dosing frequency of nitisinone in patients with HT-1. Although 1 mg/kg body weight per day divided in two daily doses is presently recommended (Orfadin^®^ summary of product characteristics), once-daily dosing is supported by the long half-life of nitisinone in plasma (median approximately 54 h = 2.3 days) (Hall et al. 2001). A retrospective study on real-life clinical practice in Europe, Turkey, and Israel showed that the dosing frequency varied from once to thrice daily with an average of twice daily (Mayorandan et al. 2014). The availability of PK data in the literature is limited, emphasizing the need of more information in this field. Since the risk of less frequent dosing is a temporary insufficient HPPD blockade with SA breakthrough, C_min_ prior to dosing was chosen as the primary PK endpoint for this study. As expected, according to pharmacokinetic principles, the mean C_min_ measured after once daily dosing was lower than after twice daily dosing. However, there was no breakthrough of SA in this study indicating that once-daily dosing was as efficacious as twice-daily dosing.

In further support of once-daily dosing is a study demonstrating that it was as effective as a multiple-dosing regimen for nine patients with HT-1 (Schlune et al. 2012). Also in favor of once-daily dosing are the preclinical studies showing a slow dissociation rate of the nitisinone-HPPD complex and slow recovery of HPPD enzyme activity, indicating that a temporary decrease in nitisinone serum concentration does not necessarily reflect a proportional loss of HPPD inhibition (Ellis et al. 1995; Lock et al. 1996).

A change in dosing frequency with maintained total dose, as in this study, does not change the overall average drug concentration in the dosage interval, only the fluctuations of drug concentration within the interval. The decrease in C_min_ is expected to be mirrored by a corresponding increase in C_max_. Correct C_max_ determination requires blood sampling over several hours, which was not considered ethical in this study due to the predominantly pediatric population. Therefore, the time for maximum concentrations (t_max_) was estimated based on previously published nitisinone steady-state levels (Olsson et al. 2015) and samples were taken 3–4 h after dosing. Unfortunately the average C_max_ was similar after switching from twice- to once-daily dosing, indicating that the timing of C_max_ sampling was suboptimal. It was therefore irrelevant to report actual fluctuations during the dosage interval (C_max_/C_min_ ratios) even though they were included as secondary endpoints.

For practical as well as ethical reasons considering very young children, this study assessed nitisinone levels from DBS (small volumes of blood and less invasive than serum samples). However, it is important to note that the nitisinone levels reported here were, due to practical constraints, not converted to corresponding serum levels as initially planned. For this reason any direct comparison to the recommended serum levels in the treatment of HT-1 would be incorrect. General awareness should be brought to the complexity of converting concentrations in DBS samples to plasma or serum concentrations since the correct conversion factor needs to be determined for each DBS method at the local laboratory if it is going to be used in therapeutic monitoring.

The low incidence of HT-1 and the fact that most patients are still pediatric pose challenges to the recruitment into clinical studies in this patient population and is a reason for the relatively low number of patients in this study, especially in the younger ages. Despite an extended enrollment period and inquiries for additional patients across Europe to fill the quota in the youngest age group (<2 years of age), no additional patients were found and it was decided to terminate the study with only two infants. The recruitment difficulty can be explained by the fact that this age group covered only 2 years while other age groups had a much wider age span. Moreover during the 2 years the infants also had to be diagnosed and have an established nitisinone treatment ongoing for several months before inclusion, leaving a very short window of recruitment opportunities for infants. Furthermore, after establishing initial treatment, parents might be hesitant to change regimen in these very young children. Thus, a total of 19 patients were enrolled in the study, not 20 as originally planned in the study protocol.

In conclusion, the results of this study contribute to the overall understanding of the flexibility of nitisinone dosing for patients with HT-1. Switching from twice-daily to once-daily dosing proved both safe and efficacious for all patients in this study and is therefore recommended. However, we cannot exclude that some individuals may benefit from more frequent dosing and a switch to once-daily dosing should ideally be carefully monitored.

## Disclosures

*Nathalie Guffon*: Coordinating investigator for the study. NG participated in several clinical trials and have institution contracts including investigators fees from Genzyme Sanofi, Shire HGT, BioMarin, and Sobi.

*Anders Bröijersén*: Employee of Sobi and holder of Sobi shares.

*Ingrid Palmgren*: Employee of Sobi and holder of Sobi shares.

*Mattias Rudebeck*: Employee of Sobi and holder of Sobi shares.

*Birgitta Olsson*: Employee of Sobi and holder of Sobi shares.

This study was fully funded by Sobi (Swedish Orphan Biovitrum AB [publ]).

## Informed Consent

All procedures followed were in accordance with the ethical standards of the responsible committee on human experimentation (institutional and national) and with the Helsinki Declaration of 1975, as revised in 2000 (5). Informed consent was obtained from all patients before being included in the study.

## Individual Author Contribution

*Nathalie Guffon* was the coordinating investigator of the study and substantially contributed to the acquisition and interpretation of data. Nathalie Guffon also actively participated in drafting the manuscript and approved of the final version to be submitted.

*Anders Bröijersén* contributed to the concept and design of the study and he also analyzed the study results and had the overall medical responsibility during the study. Anders Bröijersén also participated in drafting the manuscript, approved of the final version to be submitted and is corresponding author.

*Ingrid Palmgren* contributed to the concept and design of the study and she also analyzed the study results with particular focus on all safety aspects. Ingrid Palmgren also participated in drafting the manuscript and approved of the final version to be submitted.

*Mattias Rudebeck* contributed to the concept and design of the study and he also analyzed the study results. Mattias Rudebeck also participated in drafting the manuscript and approved of the final version to be submitted.

*Birgitta Olsson* contributed to the concept and design of the study and she also analyzed the study results with particular focus an all pharmacokinetic aspects. Birgitta Olsson also participated in drafting the manuscript and approved of the final version to be submitted.

## Abbreviations

AE: Adverse event
C_max_: Maximum concentration
C_min_: Minimum concentration
HT-1: Hereditary tyrosinemia type 1
LLOQ: Lower limit of quantification
SA: Succinylacetone
